# Screening tools for young people

**DOI:** 10.1186/1940-0640-8-S1-A27

**Published:** 2013-09-04

**Authors:** Carol Foster, Sarah Jones, Craig Jones, John Bradley, Paul Jordan

**Affiliations:** 1 Public Health Wales, Cardiff, UK

## 

In 2009, Sir Liam Donaldson, former Chief Medical Officer for England, advised parents that children “under 15 should never be given alcohol, even in small quantities”; however, underage drinking is an issue in Wales with approximately 17% of 13 year olds reporting that they have been drunk at least twice (HSBC, 2009/10). There was no significant gender gap between girls (18%) and boys (17%); which is in general agreement with a local survey (Roberts, 2011); where around one in ten secondary students report having been drunk four or more times in their life, with no significant difference in gender. The difficulty is that while there are validated and established screening tools for adults and over 16's the NICE guidelines (2010) recommend the Common Assessment Framework as the only screening tool for under 15's. This is too complex to be used as a screening aid during a brief intervention (5 minutes duration). The aim of this project is to recommend a standardised method to be used in Wales for screening young people during alcohol brief interventions. Public Health Wales (PHW) will conduct a review of the available literature to determine which methods are used internationally and to evaluate the screening tools with a view to recommending a tool for use with Health and Social partners. The review and recommendations will take place through 2013. PHW plan to ensure standardisation across Wales and to raise this at a UK level.

